# Mediating Effects of Stigma and Depressive Symptoms in a Social Media–Based Intervention to Improve Long-term Quality of Life Among People Living With HIV: Secondary Analysis of a Randomized Controlled Trial

**DOI:** 10.2196/27897

**Published:** 2021-11-09

**Authors:** Yiran Li, Yan Guo, Y Alicia Hong, Chengbo Zeng, Yu Zeng, Hanxi Zhang, Mengting Zhu, Jiaying Qiao, Weiping Cai, Linghua Li, Cong Liu

**Affiliations:** 1 Department of Medical Statistics School of Public Health Sun Yat-sen University Guangzhou China; 2 Sun Yat-sen Center for Global Health Guangzhou China; 3 Department of Health Administration and Policy College of Health and Human Services George Mason University Fairfax, VA United States; 4 South Carolina SmartState Center for Healthcare Quality Arnold School of Public Health University of South Carolina Columbia, SC United States; 5 Department of Health Promotion, Education, and Behavior Arnold School of Public Health University of South Carolina Columbia, SC United States; 6 National Center of AIDS/STD Control and Prevention Chinese Center for Disease Control and Prevention Beijing China; 7 The Jockey Club School of Public Health and Primary Care Faculty of Medicine The Chinese University of Hong Kong Hong Kong China; 8 Department of Vital Statistics Shanghai Municipal Center for Disease Control and Prevention Shanghai China; 9 Department of Infectious Diseases Guangzhou Eighth People’s Hospital, Guangzhou Medical University Guangzhou China

**Keywords:** mHealth, HIV, depressive symptoms, quality of life, structural equation model

## Abstract

**Background:**

Mobile health (mHealth) interventions have been shown to effectively improve the quality of life (QOL) among people living with HIV. However, little is known about the long-term effects of mHealth interventions.

**Objective:**

This study aims to explore the intervention mechanisms of a social media–based intervention, Run4Love, on the QOL of people with HIV over across a 9-month follow-up period.

**Methods:**

We recruited people living with HIV who were concurrently experiencing elevated depressive symptoms from an HIV outpatient clinic in South China. A total of 300 eligible participants were randomized either to the intervention group or the control group in a 1:1 ratio after they provided informed consent and completed a baseline survey. The intervention group received a 3-month WeChat-based intervention, comprising cognitive-behavioral stress management (CBSM) courses and physical activity promotion. The control group received a printed brochure on nutrition guidelines in addition to the usual care for HIV treatment. Neither participants nor the research staff were blinded to group assignment. All patients were followed at 3, 6, and 9 months. The primary outcome was depressive symptoms. Structural equation model (SEM) with longitudinal data was conducted to examine the sequential mediating effects of HIV-related stigma and depressive symptoms on the long-term intervention effects on participants’ QOL.

**Results:**

About 91.3% (274/300), 88.3% (265/300), and 86.7% (260/300) of all participants completed follow-up surveys at 3, 6, and 9 months, respectively. Results showed that the intervention had significantly improved participants' QOL at 9 months, via complete mediating effects of reduced HIV-related stigma at 3 months and decreased depressive symptoms at 6 months. No adverse events were reported.

**Conclusions:**

These findings underscore the critical roles of HIV-related stigma and depressive symptoms in an mHealth intervention with long-term effects on QOL improvements. We call for targeted mHealth interventions to improve QOL among people living with HIV, especially social media–based interventions that can address HIV-related stigma and alleviate depressive symptoms.

**Trial Registration:**

Chinese Clinical Trial Registry ChiCTR-IPR-17012606; https://www.chictr.org.cn/showproj.aspx?proj=21019

## Introduction

Owing to widely available antiretroviral therapy (ART), living with HIV has become a manageable chronic condition [1]. The 90-90-90 targets that by 2020, 90% of people living with HIV are diagnosed, 90% of those diagnosed receive sustained ART, and 90% of those on treatment are virally suppressed have been unevenly achieved in different contexts [2,3]. In addition, other challenges, such as suboptimal quality of life (QOL), high levels of stigma, and poor mental health among people living with HIV, are still prevalent [4-6]. A great body of evidence has shown that high levels of stigma and depressive symptoms could worsen existing disease conditions and lead to poor QOL [7-9]. A new goal called the fourth “90”—that 90% of people living with HIV have optimal health-related QOL—has been proposed and widely concurred [4]. Emerging research has developed multilevel effective psychosocial, behavioral, and contextualized interventions to improve the QOL among people living with HIV [10].

Although psychosocial interventions such as cognitive-behavioral stress management (CBSM) have been proved effective in improving the QOL among people living with HIV, few studies have explored the mechanisms of the effects of such interventions on QOL, especially in the long term [11-14]. Existing studies were often conducted with a pre- and post-design and a short-term follow-up, such as an 8- or 10-week follow-up [15,16]. Few studies examined the long-term intervention mechanisms of QOL improvement [17,18]. Furthermore, most of these interventions were delivered face-to-face in group settings. Given the prevalent HIV-related stigma and discrimination in many countries, including China, a large number of people living with HIV would prefer web-based or mobile-based interventions rather than face-to-face interventions due to privacy concerns.

Mobile health (mHealth) interventions have proved effective in improving QOL among people living with HIV [19-22]; however, to the best of our knowledge, no study has ever explored the intervention mechanisms on improving QOL in mHealth interventions. As mHealth interventions may reach a large number of populations with increased accessibility, convenience, identity protection, and potentially lower cost, such interventions are well suited for marginalized and stigmatized populations, such as people living with HIV [23]. With the increasing number of people living with HIV and wide coverage of smart phones, studies that explore the mechanisms of mHealth interventions on improving the QOL of people living with HIV are much needed, especially those that would examine long-term intervention mechanisms.

Previous studies have suggested that HIV-related stigma and depressive symptoms are important predictors of QOL; however, the mechanisms among these three variables have only been examined in cross-sectional studies, but not in interventional studies [7,24-27]. One cross-sectional study in Ontario, Canada, found that HIV-related stigma was associated with lower QOL, and depressive symptoms partially mediated the association between HIV-related stigma and QOL among African and Caribbean women living with HIV [24]. Another cross-sectional study in Vietnam found that people living with HIV who reported having experienced stigma were more likely to have elevated depressive symptoms and lower QOL [27]. Existing interventional studies have only explored the interventional effects on QOL and potential associated factors using regression analyses. For example, a study found that the improvement of QOL among people living with HIV was positively associated with a reduction in depressive symptoms at the 6-month follow-up in an intervention targeted on depression by regressing the change of QOL on that of depressive symptoms during the 6-month interval [28]. A similar study was also conducted on the association between improvement in QOL and reduction of HIV-related stigma [18]. Due to the limitation of the methodology, existing studies have not yet investigated the mechanisms of intervention effect on QOL.

**Figure 1:**
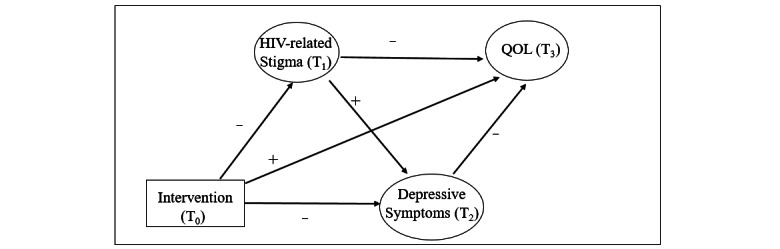
Hypothesized model of intervention, HIV-related stigma, depressive symptoms, and QOL in people living with HIV and depressive symptoms. QOL: Quality of life; T_0_: baseline; T_1_: 3-month follow-up; T_2_: 6-month follow-up; T_3_: 9-month follow-up.

To investigate the intervention mechanisms, it is important to examine the sequential relationships among influencing factors and the outcome. Structural equation model (SEM) using longitudinal data can provide a better understanding of intervention mechanisms with sequential orders. To fill the gaps of the literature, the current study aimed to explore the intervention mechanisms in the effects of a social media-based intervention, Run4Love, on QOL over 9 months. Using SEM and longitudinal data of 4-time repeated assessments, we examined the sequential relationships among HIV-related stigma, depressive symptoms, and QOL in the mHealth intervention. We hypothesized that the HIV-related stigma and depressive symptoms would play mediating roles in the intervention effects on QOL at the 9-month follow-up. Specifically, the intervention would reduce HIV-related stigma first and subsequently reduce depressive symptoms, which in turn would lead to long-term improvement in QOL among people living with HIV. The hypothesized model is illustrated in Figure 1.

## Methods

### Research Setting

This study used the data from a parallel-design randomized controlled trial (RCT). The CONSORT (Consolidated Standards of Reporting Trials) checklist was used. The study was conducted in Guangzhou, China, between September 2017 and October 2018. A total of 300 people living with HIV and depressive symptoms were recruited by the research staff from a designated hospital for HIV/AIDS treatment in Guangzhou in 2017. Guangzhou is the capital city of Guangdong province, and it is the third largest city in China. The Run4Love RCT protocol was approved by the Institutional Review Board at Sun Yat-Sen University (2018 No. 31) [29].

### Participant Eligibility

**Figure 2:**
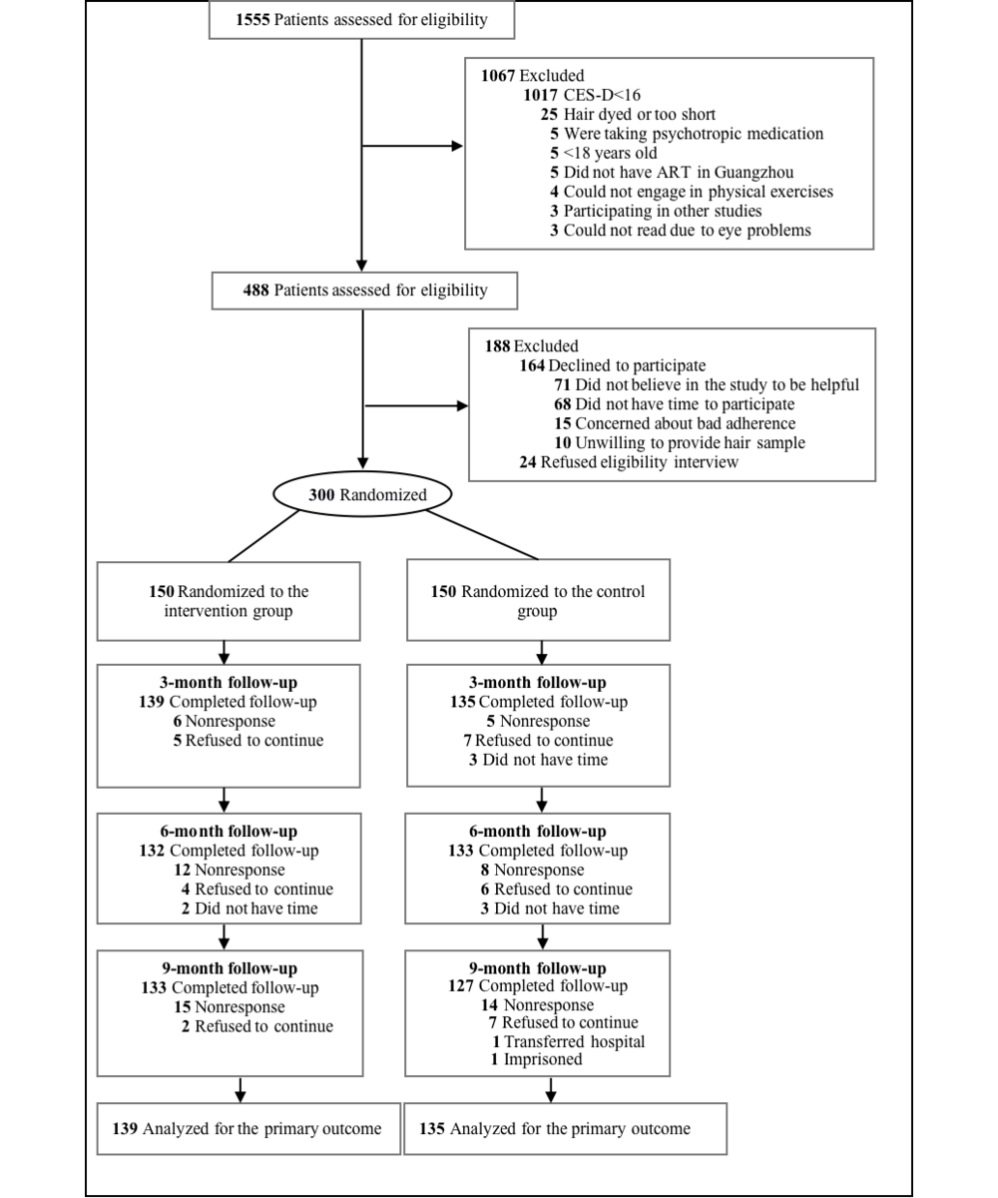
Flowchart of participant screening and recruitment. ART: antiretroviral therapy; CES-D: Center for Epidemiologic Studies-Depression.

Participants were recruited if they (1) were aged 18 years or above, (2) tested HIV-seropositive, (3) had elevated depressive symptoms (measured by the Center for Epidemiologic Studies-Depression; CES-D score ≥16), (4) were WeChat users, and (5) were willing to provide hair samples (for the purpose of measuring cortisol as a biomarker of chronic stress). Participants were excluded if they were (1) currently on psychological treatment, (2) unable to complete baseline and follow-up surveys, or (3) unable to engage in the intervention (eg, unable to read or listen to the intervention materials on WeChat or perform physical exercise due to medical or other reasons). Participants who met the eligibility criteria and were willing to participate completed a baseline survey and were randomized to the intervention or the waitlist control group with a 1:1 ratio by the research staff (Figure 2).

### Run4Love WeChat-Based Intervention

The intervention protocol has been detailed elsewhere [29]. Briefly, the Run4Love WeChat-based intervention comprised two components: adapted CBSM courses and physical activity promotion [29]. The intervention adapted the evidence-based CBSM courses into China’s context and included 9 sessions and 3 review sessions with information on meditation, muscle relaxation, and coping skills. The courses were adapted into multimedia formats, including articles, audio clips, and posters. These materials were sent through an enhanced social media platform of WeChat 3 to 5 times a week for 3 months. Physical activity promotion consisted of information on instructions and benefits of regular physical exercise. The enhanced WeChat platform had extended functions, including automatic sending of materials, patient engagement tracking on course completion, and personalized feedback on a weekly basis. In the booster session, a total of 7 most read items were redelivered to the participants in the intervention group in the 3 months following postintervention. Participants in the control group received a printed brochure on nutrition guidelines in addition to the usual care for HIV treatment.

### Sample Size

We calculated the sample size of the RCT based on the primary outcome of depressive symptoms measured by CES-D. Effect size was used to calculate between-group differences. We hypothesized an effect size of 0.4, with α=.05, β=.15, and a dropout rate of 20%, thus arriving at a sample size of 282 for the RCT. Finally, a total of 300 participants were recruited. For SEM, the recommended sample size of 5 observations per estimated parameters was considered [30]. Given a total of 46 estimated parameters in the SEM of our study, the sample size of 300 was sufficient for the analysis.

### Randomization and Masking

The research staff randomly assigned the participants to the intervention group or the control group by a computer-generated randomization list with a block size of 4 using SAS software version 9.4 (SAS Institute, Inc). By the nature of the trial design, neither the participants nor the research staff were blinded to the intervention.

### Study Outcomes

#### Overview

Individuals’ sociodemographic characteristics, HIV-related stigma, and psychosocial outcomes, including their depressive symptoms and QOL, were included in this study. Sociodemographic characteristics such as age, gender, marital status, sexual orientation, educational level, and household registration (urban and rural) were collected at baseline. Self-reported psychosocial outcomes measured at baseline and at 3-, 6-, and 9-month follow-ups were collected by researchers face-to-face via electronic questionnaires administered using a tablet device.

#### HIV-Related Stigma

HIV-related stigma was measured by the 14-item HIV Stigma Scale [31,32]. The scale measures 2 dimensions of stigma: perceived stigma and internalized stigma [31]. Perceived stigma comprises 6 items, such as “Most people think that a person who has HIV is dirty”; internalized stigma comprises 8 items, such as “I feel guilty because I have HIV.” Each item is rated on a 4-point Likert-type scale (ie, strongly disagree, disagree, agree, and strongly agree). The total score of the scale ranges from 14 to 56, with higher scores indicating higher levels of HIV-related stigma. Total scores ranging from 14 to 28, 29 to 42, and 43 to 56 are considered as low, medium, and high levels of stigma, respectively [33]. In SEM, stigma was measured by the 2 subscales (perceived and internalized stigma). Validity and reliability of the scales have been examined and established for Chinese people living with HIV [32,34]. A good reliability of HIV Stigma Scale was also shown in this study, and the Cronbach α values at baseline, 3-, 6-, and 9-month follow-ups at .92, .95, .95, and .96, respectively.

#### Depressive Symptoms

Depressive symptoms were assessed using the CES-D scale. The CES-D scale measures 4 dimensions of depressive symptoms, including depressed affect, positive affect, somatic and retarded activity, and interpersonal problems [35,36]. The scale consists of 20 items, such as “I felt depressed” and “I did not feel like eating; my appetite was poor.” Items are evaluated using a 4-point Likert scale ranging from 0 (rarely or none of the time) to 3 (most or all the time). A total score of CES-D ranges from 0 to 60, with higher scores indicating a higher level of depressive symptoms. Individuals with CES-D scores no less than 16 are considered as likely having clinical depressive symptoms [37]. The CES-D scale is one of the most widely used self-rated questionnaires on depressive symptoms and has been validated in Chinese populations, including people living with HIV [35,38,39]. In this study, CES-D showed a good reliability, with Cronbach α values reported at .77, .76, .84, and .83 at baseline, 3-, 6-, and 9-month follow-ups, respectively. In SEM, depressive symptoms were measured by the 4 dimensions of the scale.

#### QOL Assessment

QOL was assessed by World Health Organization Quality of Life HIV–short version (WHO-QOL-HIV BREF), with 31 items measuring the following 6 dimensions: physical, psychological, level of independence, social relationships, environment, and beliefs [40]. Items are rated on a 5-point Likert scale. The total score of WHO-QOL-HIV BREF ranges from 24 to 120, with higher scores indicating better QOL. Validity and reliability of the scale have been examined and established for the Chinese people living with HIV [41-43]. Cronbach α values of WHO-QOL-HIV at baseline, 3-, 6-, and 9-month follow-ups were reported at .84, .91, .94, and .94, respectively.

### Data Analysis

First, descriptive statistics were reported on demographic characteristics, baseline HIV-related stigma, depressive symptoms, and QOL. Mean and SD values were used to describe continuous variables with normal distribution, and median and IQR values, for continuous variables with skewed distribution. Frequencies and percentages were used to describe categorical variables. Kolmogorov-Smirnov and Levene tests were used for normality and homogeneity tests.

Second, group differences were examined for the outcome and mediators at 4 assessment points by independent sample *t* test for continuous variables with normal distribution and Wilcoxon rank-sum test for continuous variables with skewed distribution.

Third, bivariate analyses were performed to examine relationships between baseline demographic characteristics and QOL. Independent samples *t* tests were used to examine differences in QOL by different demographic characteristics. Pearson correlation analyses were performed to examine relationships between QOL and continuous demographic variables, such as age. Significance level of potential associations was *P*<.10. Variables significant in bivariate analyses were considered as potential confounders and controlled in the SEM.

Fourth, in SEM analyses, HIV-related stigma, depressive symptoms, and QOL were treated as latent variables and measured by their subscales [44]. SEM allows to control for measurement error of variables; thus, it yields unbiased estimates of latent variables [45,46]. To assess the goodness of fit of the measurement models, confirmatory factor analyses (CFAs) of HIV-related stigma, depressive symptoms, and QOL were performed, respectively. With satisfactory measurement models, the SEM was then conducted to examine the hypothesized mechanisms of long-term intervention effects on participants’ QOL.

Finally, a mediation model of SEM was performed to test the hypotheses of sequential mediating effects of HIV-related stigma and depressive symptoms by using longitudinal data. Such models were designed to explore the mechanisms, direct and indirect effects of the intervention, and sequential relationships [47]. Based on the associations between HIV-related stigma, depressive symptoms, and QOL in previous studies, we assigned sequential orders to the SEM variables [24,25,48-50]. Specifically, HIV-related stigma at 3-month follow-up, depressive symptoms at 6-month follow-up, and QOL at 9-month follow-up were estimated using the model. The pathways of intervention→QOL, intervention→depressive symptoms→QOL, intervention→HIV-related stigma→QOL, and intervention→HIV-related stigma→depressive symptoms→QOL were examined separately (dummy coded as 0=control group, 1=intervention group). Baseline QOL and sociodemographic characteristics that were significantly associated with QOL in bivariate analyses were controlled as covariates in the model. The statistical significance in SEM was defined at *P*<.05.

To evaluate the goodness of fit of the model, multiple indicators were used, including the chi-square statistic, Comparative Fit Index (CFI), Tucker-Lewis Index (TLI), root mean square error of approximation (RMSEA), and standardized root mean square residual (SRMR). A smaller chi-square value indicates better model fit. Moreover, CFI ≥0.95, TLI >0.90, RMSEA ≤0.06, and SRMR ≤0.08 indicate good model fit [51,52]. Descriptive statistics, bivariate statistics, and correlation analyses were performed using SAS version 9.4 (SAS Institute, Inc). CFA and SEM were tested by robust maximum likelihood method (estimator=MLR in Mplus) and performed using Mplus version 7.0 [53,54].

## Results

### Baseline Characteristics

The Run4Love program recruited 300 participants; their baseline characteristics are described in Table 1. Participants’ mean age was 28.3 (SD 5.8) years. Most of the participants were male (277/300, 92.3%), nonheterosexual (245/300, 81.7%), not married (262/300, 87.3%), and well educated (high school and above: 182/300, 60.7%). The mean scores of HIV-related stigma, CES-D, and QOL were 37.5 (SD 7.6), 24.1 (SD 6.6), and 77.0 (SD 9.2), respectively, at baseline. A majority (213/300, 71.0%) of the participants had a moderate level of stigma, whereas 20.3% (61/300) had high level and 8.7% (26/300) had low level of stigma.

**Table 1:** Baseline characteristics and outcomes of study participants by intervention and control group in the Run4Love program (n=300).

Baseline characteristics		Total (n=300)	Intervention group (n=150)	Control group (n=150)	*P* value
Age (years), mean (SD)		28.3 (5.8)	28.0 (5.8)	28.6 (5.9)	.39^a^
Male, n (%)		277 (92.3)	142 (94.7)	135 (90)	.13^b^
Educational level above high school, n (%)		182 (60.7)	98 (65.3)	84 (56)	.10^b^
Homosexual, bisexual, or uncertain, n (%)		245 (81.7)	130 (86.7)	115 (76.7)	.03^b^
Married, n (%)		38 (12.7)	18 (12)	20 (13.3)	.73^b^
Employed, n (%)		251 (83.7)	123 (82)	128 (85.3)	.17^b^
Depressive symptoms, mean (SD)		24.1 (6.6)	23.9 (6.4)	24.3 (6.9)	.68^a^
QOL^c^, mean (SD)		77.0 (9.2)	77.4 (9.0)	76.6 (9.4)	.44^a^
**HIV-related stigma, mean (SD)**		37.5 (7.6)	37.1 (7.7)	38.0 (7.5)	.31^a^
	Low, n (%)	26 (8.7)	15 (10)	11 (7.3)	.36^b^
	Medium, n (%)	213 (71)	109 (72.7)	104 (69.3)	
	High, n (%)	61 (20.3)	26 (17.3)	35 (23.3)	

^a^Based on independent-samples *t* test.

^b^Based on chi-square test.

^c^QOL: quality of life.

The proportion of homosexual, bisexual, or sexual orientation-uncertain participants in the control group was slightly lower than that in the intervention group (115/150, 76.7% vs 130/150, 86.7%; *P*=.03). Other demographic characteristics and psychosocial outcomes (HIV-related stigma, depressive symptoms, and QOL) were balanced between the two groups at baseline.

### Changes in QOL and Mediating Variables Over Time

The proportion of participants who lost to follow-ups was less than 15% over 9 months. Of all participants, 91.3% (274/300; n=139 in the intervention group; n=135 in the control group), 88.3% (265/300; n=132 in the intervention group; n=133 in the control group), and 86.7% (260/300; n=133 in the intervention group; n=127 in the control group) completed the follow-up surveys at 3, 6, and 9 months. The characteristics of the participants who lost to follow-ups were not significantly different from the remaining participants.

As shown in Table 2, the Run4Love social media-based intervention had significant effects on QOL and potential mediating variables over 9 months. Repeated measurements of the outcome variable (QOL) and potential mediators, including HIV-related stigma and depressive symptoms at 4 assessment points for the intervention and control groups, are shown in Table 2. The intervention significantly reduced HIV-related stigma and depressive symptoms, and improved QOL in intervention group participants compared to control group participants at 3-, 6-, and 9-month follow-ups. No adverse events were reported.

**Table 2:** Repeated measurements of QOL^b^ and potential mediators in the Run4Love program.

Variables			Intervention group, mean (SD)		Control group, mean (SD)		Effect size (95% CI)		*t* test (*df*)		*P* value	
**HIV-related stigma**												
	Baseline (T_0_)	37.10 (7.67)		37.99 (7.54)		N/A^a^		−1.01 (298)		.31		
	3 months (T_1_)	34.28 (9.19)		37.50 (8.27)		0.33 (0.09-0.57)		−3.05 (272)		.003		
	6 months (T_2_)	34.30 (8.52)		37.35 (9.92)		0.27 (0.03-0.51)		−2.69 (263)		.008		
	9 months (T_3_)	33.98 (9.01)		37.79 (9.99)		0.37 (0.12-0.62)		−3.23 (258)		.001		
**Depressive symptoms**												
	Baseline (T_0_)	23.93 (6.39)		24.25 (6.86)		N/A		−0.42 (298)		.68		
	3 months (T_1_)	17.87 (9.44)		23.85 (10.11)		0.66 (0.42-0.90)		−5.06 (272)		<.001		
	6 months (T_2_)	17.60 (10.06)		24.11 (11.42)		0.63 (0.38-0.88)		−4.93 (263)		<.001		
	9 months (T_3_)	17.86 (10.72)		23.43 (11.45)		0.51 (0.26-0.76)		−4.05 (258)		<.001		
**QOL^b^**												
	Baseline (T_0_)	77.43 (9.03)		76.59 (9.43)		N/A		0.78 (298)		.44		
	3 months (T_1_)	82.54 (12.03)		76.63 (11.08)		0.55 (0.31-0.79)		4.23 (272)		<.001		
	6 months (T_2_)	83.51 (12.88)		76.32 (12.96)		0.68 (0.43-0.93)		4.53 (263)		<.001		
	9 months (T_3_)	83.48 (13.17)		76.54 (13.34)		0.52 (0.27-0.77)		4.22 (258)		<.001		

^a^N/A: not applicable.

^b^QOL: quality of life.

### Correlations Between Demographics and QOL

Table 3 shows the correlations between demographic characteristics and QOL at baseline. Bivariate analyses indicated that gender, sexual orientation, education, marital status, and household registration were significantly associated with QOL among people living with HIV and depressive symptoms at baseline. Specifically, those who were male, nonheterosexual, and unmarried; had a higher education level; or had an urban household registration had better QOL at baseline. These factors were included as controlling covariates in the SEM.

**Table 3:** Bivariate analyses of quality of life (QOL) and demographic characteristics at baseline among people living with HIV and depressive symptoms in the Run4Love program.

Demographics		QOL	*t* value (*df*)	*P* value
Age (years)		N/A^a^	N/A	.76^b^
**Gender**			1.76 (25)	.09^c^
	Male, n (%)	77.33 (9.02)		
	Female, n (%)	73.22 (10.9)		
**Sexual orientation**			−1.95 (72)	.055^c^
	Heterosexual, n (%)	74.58 (10.49)		
	Homosexual, bisexual, or uncertain, n (%)	77.56 (8.85)		
**Education**			−3.64 (234)	<.001^c^
	Less than or up to high school, n (%)	74.61 (9.51)		
	Above high school, n (%)	78.57 (8.72)		
**Marital status**			2.02 (50)	.049^c^
	Unmarried, n (%)	77.40 (9.26)		
	Married, n (%)	74.34 (8.65)		
**Employment status**			−1.19 (274)	.23^c^
	Unemployed, n (%)	76.25 (8.67)		
	Employed, n (%)	77.52 (9.57)		
**Household registration**			−2.42 (224)	.02^c^
	Rural, n (%)	76.01 (8.97)		
	Urban, n (%)	78.67 (9.45)		

^a^N/A: not applicable.

^b^Based on Pearson correlation analysis (*r*=.02).

^c^Based on independent samples *t* test.

### Measurement Model

**Figure 3:**
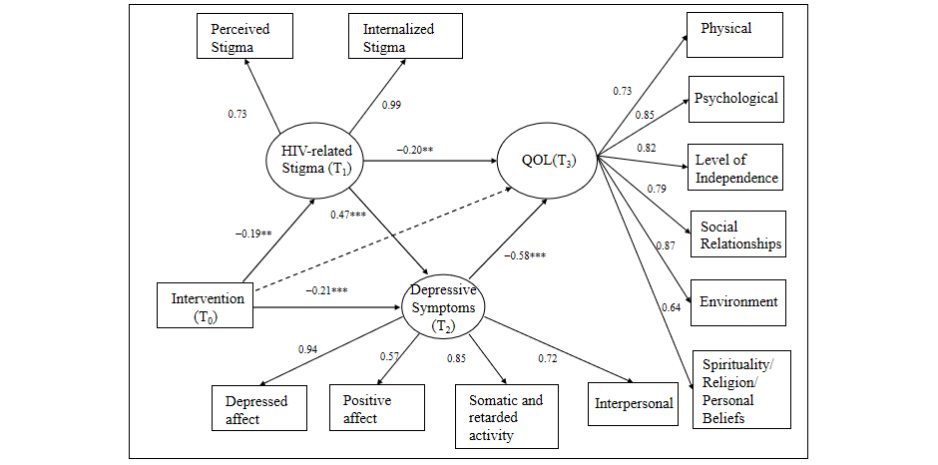
Estimation of the structural equation model. Covariates including gender, sexual orientation, education, marital status, and household registration were controlled in the structural equation model. ** *P*<.01, *** *P*<.001. QOL: Quality of life; T_0_: baseline; T_1_: 3-month follow-up; T_2_: 6-month follow-up; T_3_: 9-month follow-up.

CFA indicated that the measurement models of HIV-related stigma, depressive symptoms, and QOL yielded good model fit. Indices of the measurement models were reported in Table 4. All factor loadings of each scale were significant at *P*<.05 level. Results of the standardized factor loadings are shown in Figure 3.

**Table 4:** Confirmatory factor analyses indices of HIV-related stigma, depressive symptoms, and quality of life.

	Chi-square (*df*)	CFI^a^	TLI^b^	RMSEA^c^	SRMR^d^
HIV-related stigma	0 (0)	1.00	1.00	<0.01	<0.01
Depressive symptoms	3.04 (2)	1.00	1.00	0.04	0.01
QOL^e^	6.47 (8)	1.00	1.00	<0.01	<0.01
Good model fit	N/A	≥0.95	≥0.90	≤0.06	≤0.08

^a^CFI: Comparative Fit Index.

^b^TLI: Tucker-Lewis Index.

^c^RMSEA: root mean square error of approximation.

^d^SRMR: standardized root mean square residual.

^e^QOL: quality of life.

### Structural Model

The structural model showed a satisfactory model fit (*X^2^*_138_=240.4; *P*<.001; CFI=0.93; TLI=0.92; RMSEA=0.06; SRMR=0.06). Demographic characteristics, including gender, sexual orientation, education, marital status, and household registration, were controlled as covariates in the model.

Except for the pathway from intervention to QOL, other pathways were significant, which means that the Run4Love intervention had complete mediation effects on QOL via stigma and depressive symptoms. Standardized regression coefficients for the model are reported in Figure 3 and Table 5. Results indicated that the intervention reduced participants’ stigma at the 3-month follow-up (standardized *β*=−.19, *P*=.001), which was positively associated with depressive symptoms at the 6-month follow-up (standardized *β*=.47, *P*<.001) and negatively associated with QOL at 9-month follow-up (standardized *β*=−.20, *P*=.005). The intervention also reduced depressive symptoms (standardized *β*=−.21, *P*<.001), which consequently resulted in improved QOL at 9 months (standardized *β*=−.58, *P*<.001). The pathway from depressive symptoms to QOL had the strongest effect size in the mediation model (standardized *β*=−.58, *P*<.001). There were complete mediation effects of the intervention on QOL via stigma and depressive symptoms. Specifically, the pathway through depressive symptoms alone accounted for the largest mediation effect size of 57.1% (0.12/0.21). The path through stigma→depressive symptoms and stigma alone accounted for 23.8% (0.05/0.21) and 19% (0.04/0.21) of the total effect size on long-term QOL, respectively.

**Table 5:** Pathway coefficients of the structural equation model.

Pathways	Estimate *β*	Standardized estimate *β*	95% CI	SE	*P* value
Intervention→stigma	−2.11	−.19	−3.37 to −0.85	0.64	.001
Intervention→depressive symptoms	−2.05	−.21	−3.11 to −0.99	0.54	<.001
Intervention→QOL^a^	.21	.05	−0.18 to 0.60	0.05	.28
Stigma→depressive symptoms	.41	.47	0.30 to 0.52	0.06	<.001
Stigma→QOL	−.08	−.20	−0.14 to −0.02	0.03	.005
Depressive symptoms→QOL	−.26	−.58	−0.34 to −0.18	0.04	<.001
Total effects from intervention to QOL	1.13	.26	0.60 to 1.66	4.21	<.001
Direct effect	.21	.05	−0.18 to 0.60	0.20	.28
Indirect effects	.92	.21	0.53 to 1.30	0.20	<.001
Intervention→stigma→QOL	.17	.04	0.00 to 0.33	0.08	.048
Intervention→depressive symptoms→QOL	.53	.12	0.23 to 0.82	0.15	<.001
Intervention→stigma→depressive symptoms→QOL	.22	.05	0.07 to 0.38	0.08	.005

^a^QOL: quality of life.

## Discussion

### Principal Findings

To our knowledge, this study is among the first efforts to explore the potential causal mechanisms of long-term improvement in QOL in an mHealth intervention using longitudinal data. The findings revealed significant mediating roles of stigma and depressive symptoms in improving long-term QOL for the mHealth intervention participants. Specifically, the long-term (9-month follow-up) intervention effect on the improvement of QOL was entirely mediated by the reduction of stigma in the short term (3 months) and reduction of depressive symptoms in the mid term (6 months).

Although several studies have explored the mechanisms of psychosocial interventions, the mechanisms of QOL improvement in the long term remain understudied. For example, studies on mind-body therapies have found that mindfulness might serve as a potentially important mechanism for patients’ QOL improvement in such interventions [16,55]. However, literature examining the mechanisms of QOL improvement in psychosocial interventions are scarce, especially in the long term. In addition, methods employed in previous studies such as repeated measures analyses of covariance and latent growth curve model could not identify the sequential and causal relationships when exploring intervention mechanisms [16,17,56]. Our study contributed to the literature by affirming the sequential and potential causal relationships between HIV-related stigma, depressive symptoms, and QOL, which have not been reported in previous studies. Furthermore, only a small number of studies used longitudinal data, of which, to the best of our knowledge, none examined the sequential relationships [16,17,56]. In this study, the use of SEM with longitudinal data allowed us to assign mediators and the outcomes in chronological order, thus shedding light on the potential causal relationships of the intervention mechanisms.

### Practical Implications

Given the critical roles of stigma and depressive symptoms in improving long-term QOL, it is important to address stigma and alleviate depressive symptoms in interventions aiming for QOL improvement among people living with HIV. There are several effective ways to reduce HIV-related stigma and to alleviate participants’ depressive symptoms as suggested in the literature and shown in this study. First, the intervention content should be evidence-based in reducing HIV-related stigma and alleviating depressive symptoms [5,12]. The intervention materials of the Run4Love program were adapted from the evidence-based and theory-guided CBSM courses, which have been proved effective in reducing stigma and depressive symptoms among people living with HIV [12,57,58]. The core elements of the CBSM courses, such as stress management and coping skills, were preserved and adapted into multimedia formats. The participants in the intervention groups had a moderate level of patient engagement. The cumulative completion rates were 50.6%, 51.5%, and 50.8% at 1, 2, and 3 months, respectively, in the intervention group, which were comparable to other mHealth interventions [59]. The positive relationship between patient engagement and health outcomes has been confirmed in our previous study [59]. Therefore, evidence-based intervention content, rigorous design, and implementation might have contributed to the significant reduction of HIV-related stigma and depressive symptoms in the intervention group.

Second, another effective way to reduce stigma is through social contact; this is one of the most effective strategies to reduce stigma, provide social support, and alleviate depressive symptoms [5,60,61]. Comparatively, mHealth interventions provide varied forms of social contact with better convenience and privacy than face-to-face interventions [62,63]. For example, anonymous virtual communities, such as community message board and web-based forums, serve as easy and secure access for people living with HIV to interact with peers and receive social support [62,63]. Earlier studies suggested that mass media communication or supportive virtual groups could be incorporated in interventions to mitigate stigma among people living with HIV [62,64]. Such web-based communities and connections are much needed by people living with HIV because of persistent stigma—both perceived and internalized—against HIV and people affected by HIV, as well as the consequent fear of discrimination and social isolation [65,66]. In addition, mHealth interventions allow alternative web connections (eg, human-machine interactions) to incentivize and engage participants. For example, the Run4Love program incorporated automatic weekly feedback on each participant’s completion status of the intervention materials, with personalized verbal incentives. Such personalized and patient-centered human-machine interaction is a unique advantage of mHealth interventions. Future mHealth interventions for people living with HIV need to incorporate social contact in various forms as an essential component to address HIV-related stigma and decrease social isolation, so as to reduce depressive symptoms and achieve long-term improvement in people living with HIV’s QOL.

Third, since HIV-related stigma is a multidimensional aspect, public policies and coordinated efforts are needed to reduce HIV-related stigma at multiple levels [67,68]. At the societal level, public policies and effective interventions targeting on stigma reduction, HIV testing, ART provision, and social empowerment are needed to address HIV-related stigma among people living with HIV [69]. Community-based support services and educational campaigns should be prioritized to reduce stigma and discrimination [70]. For example, in China, many celebrities, including the First Lady Peng Liyuan, joined the public campaign to reduce discrimination against people living with HIV and promote HIV testing and treatment [71,72]. At the health care level, Voluntary Counseling and Testing program has been instituted at the Centers for Disease Control and Prevention (CDC) and local hospitals [73]. At the individual level, a number of intervention programs focused on stigma reduction, problem-solving skills, and psychological support have been implemented [68].

### Limitations

There are some limitations to this study. First, we only focused on HIV-related stigma and depressive symptoms as two important mediators; however, other variables, such as stress, social support, and self-efficacy, might also be potential mediating factors and were not included in this study. Future studies are needed to explore the mediating effects of these factors to better understand the mechanisms of social media-based mHealth interventions. Second, the measurements of stigma, depressive symptoms, and QOL were self-reported and thus might introduce potential recall bias. However, SEM analysis has already considered the measurement errors of these psychosocial variables and therefore provided more reliable model estimations. Third, since the participants were recruited from one hospital in an urban setting and the sample comprised mostly male participants, the generalizability of the study findings to other patients or geographical locations needs to be taken with caution, especially for women and those living in rural areas.

### Conclusions

In conclusion, this study is one of the first efforts to examine the potential causal mechanisms of an mHealth intervention in long-term QOL improvement by using longitudinal data and SEM. We found that the long-term intervention effect on QOL improvement was entirely mediated by the reduction of stigma in the short term and reduction of depressive symptoms in the mid term. The findings underscore the critical roles of reducing HIV-related stigma and depressive symptoms in mHealth interventions targeting QOL improvements among people living with HIV. We call for targeted mHealth interventions to improve long-term QOL among people living with HIV, especially social media-based interventions that can reduce HIV-related stigma and alleviate depressive symptoms.

## Abbreviations

ART: antiretroviral therapy
CBSM: cognitive-behavioral stress management
CDC: Centers for Disease Control and Prevention
CES-D: Center for Epidemiologic Studies-Depression
CFA: confirmatory factor analysis
CFI: Comparative Fit Index
mHealth: mobile health
QOL: quality of life
RMSEA: root mean square error of approximation
SEM: structural equation model
SRMR: standardized root mean square residual
TLI: Tucker-Lewis Index
WHO-QOL-HIV BREF: World Health Organization Quality of Life HIV–Short Version
